# ﻿More hidden diversity in a cryptic species complex: a new subspecies of *Leptideasinapis* (Lepidoptera, Pieridae) from Northern Iran

**DOI:** 10.3897/compcytogen.17.102830

**Published:** 2023-05-04

**Authors:** Vazrick Nazari, Vladimir A. Lukhtanov, Alireza Naderi, Zdenek Faltýnek Fric, Vlad Dincă, Roger Vila

**Affiliations:** 1 Department of Biology, University of Padova, Padova, Italy University of Padova Padova Italy; 2 Department of Karyosystematics, Zoological Institute of Russian Academy of Science, Universitetskaya nab. 1, 199034 St. Petersburg, Russia Zoological Institute of Russian Academy of Science St. Petersburg Russia; 3 National Natural History Museum & Genetic Resources, Tehran, Iran National Natural History Museum & Genetic Resources Tehran Iran; 4 Department of Biodiversity and Conservation Biology, Institute of Entomology, Biology Centre of the Czech Academy of Sciences, České Budějovice, Czech Republic Institute of Entomology, Biology Centre of the Czech Academy of Sciences České Budějovice Czech Republic; 5 Ecology and Genetics Research Unit, University of Oulu, Oulu, Finland University of Oulu Oulu Finland; 6 Research Institute of the University of Bucharest (ICUB), University of Bucharest, Bucharest, Romania University of Bucharest Bucharest Romania; 7 Institut de Biologia Evolutiva (CSIC – Universitat Pompeu Fabra), Barcelona, Spain Universitat Pompeu Fabra Barcelona Spain

**Keywords:** allopatry, butterflies, DNA barcoding, Palearctic, taxonomy, Wood White

## Abstract

A new subspecies of *Leptideasinapis* from Northern Iran, discovered by means of DNA barcoding, is described as *Leptideasinapistabarestana***ssp. nov.** The new subspecies is allopatric with respect to other populations of *L.sinapis* and is genetically distinct, appearing as a well-supported sister clade to all other populations in COI-based phylogenetic reconstructions. Details on karyotype, genitalia, ecology and behaviour for the new subspecies are given and a biogeographical speciation scenario is proposed.

## ﻿Introduction

The cryptic diversity within the *Leptideasinapis* (Linnaeus, 1758) complex progressively came to light in recent history (Réal 1988) with the discovery of differences in genitalic morphology (Lorković 1993) and allozyme markers (Martin et al. 2003) between *L.sinapis* and *L.reali* Reissinger, 1989. It is considered one of the first documented cases of cryptic species in Europe. Since then, numerous studies have revealed a plethora of new information on the mechanisms of speciation within this species complex (Mazel 2005; Bolshakov 2006; Friberg et al. 2008), including the presence of an additional widespread hidden taxon, *L.juvernica* Williams, 1946 (e.g. Dincă et al. 2011, 2013, 2021; Lukhtanov et al. 2011; Šíchová et al. 2015, 2016; Vodă et al. 2015; Shtinkov et al. 2016; Talla et al. 2017, 2019a, b; Leal et al. 2018; Platania et al. 2020; Yoshido et al. 2020; Näsvall et al. 2021). Despite the explosion of interest in this group, many regions of Eurasia where *Leptidea* species occur are still not well sampled or studied. The new subspecies described in this paper was discovered accidentally in the course of a genetic investigation in order to determine whether any of the populations of *L.sinapis* in Iran belong to the related cryptic species *L.juvernica*.

## ﻿Materials and methods

Fourteen Iranian specimens from various disjunct populations in NW and N Iran were selected ex. coll. A. Naderi (Tehran) and W. ten Hagen (Germany) and their legs were submitted for DNA barcoding. Samples were processed in the Center for Biodiversity Genomics in Guelph, Ontario, Canada using standard protocols and LepF/LepR primers, supplemented by failure-tracking with mini-primers (mLepF and mLepR) (Hajibabaei et al. 2006). Eleven additional samples from Javaherdeh (VLU396-VLU405, RVcoll10C196) sequenced in 2012 were later added to the dataset. The majority of these sequences were full length barcodes (658 bp). An additional specimen from Javaherdeh included later in our analysis (MR ZF 449) was isolated using the Geneaid Blood and Tissue kit and sequenced in the Czech Republic using RON-HCO primers, and thus only partially overlaps (420 bp) with the standard barcode region. Thirty-six new barcode sequences were submitted to GenBank (Accessions OQ359842–OQ359877). In addition to the sequences pertaining to the new taxon, a selection of 80 other samples from previous studies (Lukhtanov et al. 2011; Dincă et al. 2013; Shtinkov et al. 2016) representing various haplotypes of *L.sinapis* and several other species of *Leptidea* was used to conduct the analyses in this study (Suppl. material 1). All records are publicly available in the BOLD dataset “DS-SINIRAN” (https://doi.org/10.5883/DS-SINIRAN).

A Maximum Likelihood (ML) tree was generated with PHYML online (Guindon and Gascuel 2003) using the AIC criterion and 100 bootstrap replicates. The best-fit model selected by PHYML for the combined dataset (GTR + *Γ* + *I*) was further corroborated by IQ-TREE (Nguyen et al. 2015), and parameters from this model were used to conduct a Bayesian analysis in MRBAYES 3.2.6 (Ronquist et al. 2012). The MCMC analysis was allowed to run for 10,000,000 generations until stationary was reached. Convergence of parameters after the exclusion of the burnin phase was tested using TRACER 1.7.1 (Rambaut et al. 2018). Trees were edited using FIGTREE 1.4.4 (Rambaut 2018). Genetic distances were calculated using the Maximum Composite Likelihood model in MEGA 11.0.8 (Tamura et al. 2021). A haplotype diagram only including *L.sinapis*, *L.juvernica* and *L.reali* was constructed in TCS 1.21 (Clement et al. 2000), with a 95% confidence limit for parsimony. Shorter barcode fragments or those with ambiguous bases were excluded from haplotype analyses.

Male genitalia were examined following maceration in 10% potassium hydroxide (KOH) for 15 minutes at 95 °C, dissection and cleaning under a stereomicroscope and storage in tubes with glycerol. Male genitalia were photographed in a thin layer of 30% ethanol (without being pressed under a cover slip), using a Carl Zeiss Stemi 2000-C stereomicroscope equipped with a CMEX PRO-5 DC.5000p digital camera (RV) or a Leica DFC450 digital camera (ZFF). Care was taken to arrange the measured structures parallel to the focal plane of the stereomicroscope in order to minimize the measurement error. Measurements were performed based on digital photographs using the AxioVision software (Carl Zeiss MicroImaging GmbH). Eight specimens were analysed and the dataset was combined with data from Dincă et al. (2011) (135 specimens). We measured three elements of the male genitalia: phallus length (PL), saccus length (SL) and vinculum width (VW), known to be the most informative for differentiating *Leptidea* species (e.g. Dincă et al. 2011; Shtinkov et al. 2016) (Suppl. material 2). Bivariate scatterplots were generated using VW as a size variable (Shtinkov et al. 2016).

Chromosome preparations were made for ten adult males representing the population from Javaherdeh (field codes VLU396-VLU405) and were processed as previously described (Vershinina and Lukhtanov 2010). Briefly, gonads were removed from the abdomen and placed into freshly prepared fixative (3:1; 96% ethanol and glacial acetic acid) directly after capturing the butterfly in the field. Testes were stored in the fixative for 3–36 months at +4 °C. Then the gonads were stained in 2% acetic orcein for 30–60 days at +18–20 °C. Metaphase II (MII) and mitotic plates were examined using the original two-phase method of chromosome analysis (Lukhtanov et al. 2020a). Abbreviation “ca” (circa) means that the count was made with approximation due to overlapping of some chromosomes or due to difficulties in distinguishing between chromosome bivalents and trivalents. Images were edited in open source software GIMP 2.10.32 (The GIMP Development Team 2019) and Inkscape X11 (Inkscape Project 2020). Map was created using Simplemappr (Shorthouse 2010).

## ﻿Results

**Figure 1.**
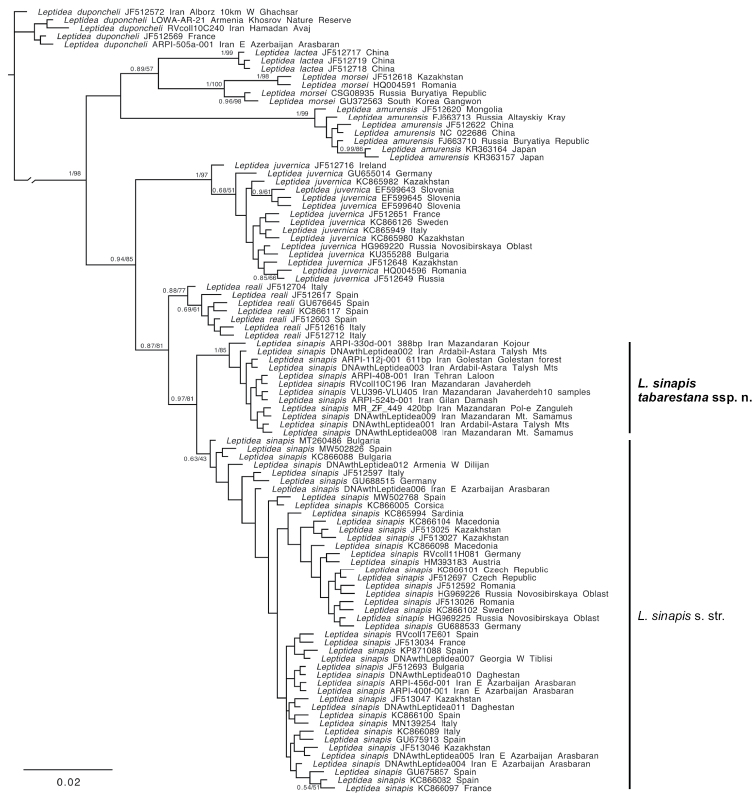
Bayesian phylogeny of *Leptidea* COI barcodes. Node support values (Bayesian Posterior Probebilities / ML bootstrap) are shown only for supported nodes. All sequences are 658 bp in length unless indicated otherwise.

**Figure 2.**
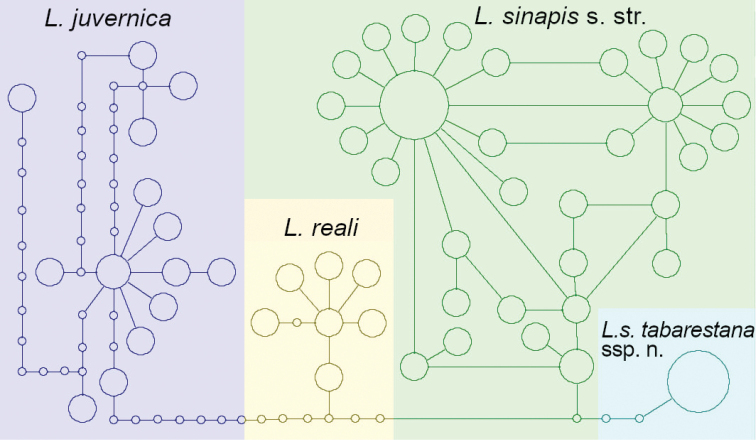
TCS haplotype network for *L.sinapis*, *L.reali* and *L.juvernica*.

None of the barcoded Iranian specimens belonged to *L.juvernica.* Specimens from the Iranian province of East Azerbaijan (Arasbaran) showed several haplotypes identical to those of the common and widespread Eurasian *L.sinapis*; however, samples collected across the Alborz mountains from Talesh to NE Iran represented a unique and well-supported COI clade that appeared as sister to a weakly-supported clade containing all other *L.sinapis* (Figs 1, 2). A comparison of average uncorrected pairwise distances between this new lineage and other *Leptidea* species showed that it is indeed genetically closer to *L.sinapis* (average: 0.74%; range: 0.42%–1.76%) and further from all the other *Leptidea* (Table 1).

**Table 1.** Average uncorrected *p*-distances (in % of the COI barcoding region) and standard deviation between *Leptidea* taxa.

	*L.duponcheli*	*L.lactea*	*L.morsei*	*L.amurensis*	*L.juvernica*	*L.reali*	*L.s.sinapis*	*L.s.tabarestana*
*L.duponcheli* (n=5)	0.27 ± 0.13							
*L.lactea* (n=3)	5.80 ± 0.13	0.00 ± 0.00						
*L.morsei* (n=4)	5.94 ± 0.26	2.33 ± 0.26	0.71 ± 0.44					
*L.amurensis* (n=7)	7.40 ± 0.14	4.23 ± 0.09	3.75 ± 0.13	0.26 ± 0.16				
*L.juvernica* (n=15)	6.29 ± 0.20	2.51 ± 0.16	3.39 ± 0.24	3.97 ± 0.15	0.30 ± 0.13			
*L.reali* (n=7)	5.35 ± 0.16	2.33 ± 0.13	2.96 ± 0.25	3.79 ± 0.15	1.75 ± 0.16	0.21 ± 0.07		
*L.s.sinapis* (n=44)	5.72 ± 0.18	2.71 ± 0.18	3.05 ± 0.21	3.74 ± 0.21	1.97 ± 0.21	0.92 ± 0.15	0.24 ± 0.11	
***L.s.tabarestana*** (n=21)	5.69 ± 0.18	2.76 ± 0.16	2.78 ± 0.25	4.02 ± 0.13	2.00 ± 0.17	0.96 ± 0.19	**0.74** ± **0.20**	0.02 ± 0.05

**Figure 3.**
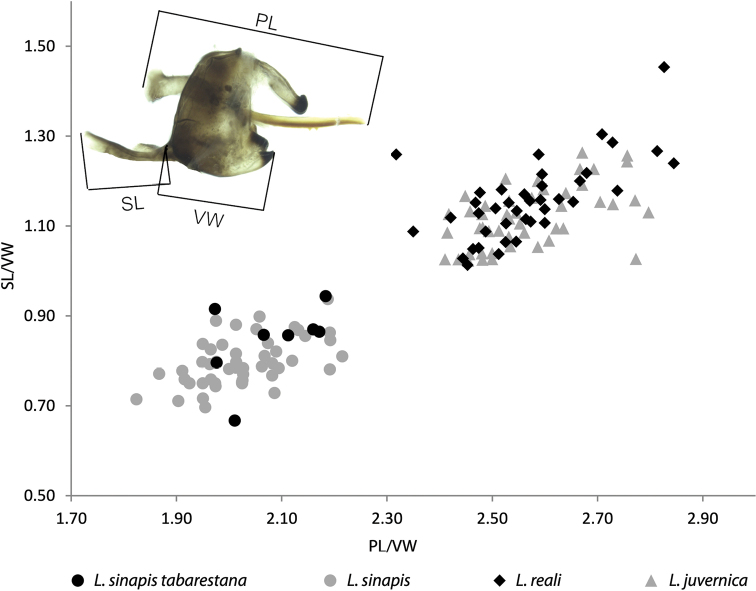
Bivariate scatterplot based on male genitalia morphometry (phallus length, PL; saccus length, SL), using vinculum width (VW) as a size variable. *L.s.tabarestana* ssp. nov. overlaps broadly with *L.s.sinapis* s. str., however it is distinct from *L.juvernica* and *L.reali.* Inset: Male genitalia of *L.sinapistabarestana* ssp. nov. (specimen MR ZF 449), showing the variables measured.

The genitalia of the eight specimens analysed belonging to the above-mentioned COI lineage showed broad overlap with other specimens of *L.sinapis* and a certain degree of variability, despite their fairly restricted geographic origin (Fig. 3). Based on the three characters measured (PL, SL, VW), the male genitalia also indicated a close similarity to *L.sinapis*, with respect to which we did not notice any significant differences.

Considering the allopatric distribution of the new taxon with respect to *L.sinapis*, its similar genitalia, and the fact that the new taxon appears to be genetically closer and phylogenetically sister to the rest of *L.sinapis* specimens, here we describe it as a new subspecies of *L.sinapis*:

### ﻿ Leptidea sinapis ssp. tabarestana

Taxon classificationAnimaliaLepidopteraPieridae

Nazari, Lukhtanov et Naderi ssp. nov.

1C5BE43F-D095-58E1-BC85-350DBEE92A76

https://zoobank.org/BED12A6B-C1D3-4897-8D40-A955333D6C7C

Fig. 4a–i

#### Type material.

***Holotype*.** ♂ [white label] “330d= Mazandaran- E Kojour-/Kodir – 1000 m – 2.Jul.[20]10- / leg. A.R. Naderi”; [red label] “Holotype/ *Leptideasinapistabarestana* / Nazari, Lukhtanov & Naderi 2023”. BOLD Sample ID: ARPI-330d-001; Deposited in coll. National Natural History Museum & Genetic Resources of Department of Environment, Tehran, Iran.

***Paratypes*. Gilan**: 1♂ Damash, 1200m, 26.III.2021, leg. et coll. A.R. Naderi (ARPI-524b-001, AR# 254); 1♀ Khoshkab, rd. Siyahkal-Deylaman, 02.VII.1990, leg. et coll. Harandi. **Ardabil/Gilan**: 2♂♂ 1♀ Paß Ardabil-Astara (Paßhöhe, W Tunnel), 1600m, 10.V.2010, W. ten Hagen. **Tehran**: 1♀ Laloon, 2000–2200 m, 30.VIII.2013, leg. et coll. A.R. Naderi (ARPI-408-001, AR# 186). **Mazandaran**: 1♂ Chalus road, Yush road, 40 km from Pole Zangooleh, 2400 m, 4.VII.1997, leg. & coll. A.R. Naderi (AR# 58); 2♂♂ Galanderoud, 1000 m, 13.VII.07, leg. & coll. A.R. Naderi; 1♂ Siahkal, 03.VII.1990, leg. et coll. Harandi; 1♂ Pol-e Zanguleh – Baladeh Rd, W of Minak, 36.2254°N, 51.58409°E, 15.V.2016, leg. & coll. Z. F. Fric, Biology Centre CAS, Institute of Entomology (IECA) (MR ZF 449); 10♂♂ Javaherdeh (Jirkooh), 36.866, 50.506, 24.VII.2011, leg. V. Lukhtanov & N. Shapoval, in Institut de Biologia Evolutiva (CSIC-UPF), Butterfly Diversity and Evolution Lab (VLU396-VLU405); 43♂♂, 12♀♀ *ibid*, in coll. Zoological Institute of Russian Academy of Sciences; 1♂, Javaherdeh (Samamus Mt.), 14.VIII.2010, leg. V.V. Tshikolovets, in Institut de Biologia Evolutiva (CSIC-UPF), Butterfly Diversity and Evolution Lab (RVcoll10C196); 1♂ Samamus Mt., 15 km S Ramsar, 1350 m, 8.VIII.2003, leg. W. ten Hagen; 1♂ Samamus Mt., S Rudbar, N Javaherdeh, 1500 m, 21.VI.2006, leg. W. ten Hagen; 1♂ Samamus Mt., 2800 m, 29.V.2009, leg. et coll. Harandi. **Golestan**: 1♂ Golestan Forest, 800–1000 m, 13.V.2001, leg. & coll. A.R. Naderi (112j, AR# 185).

#### Description.

**Figure 4.**
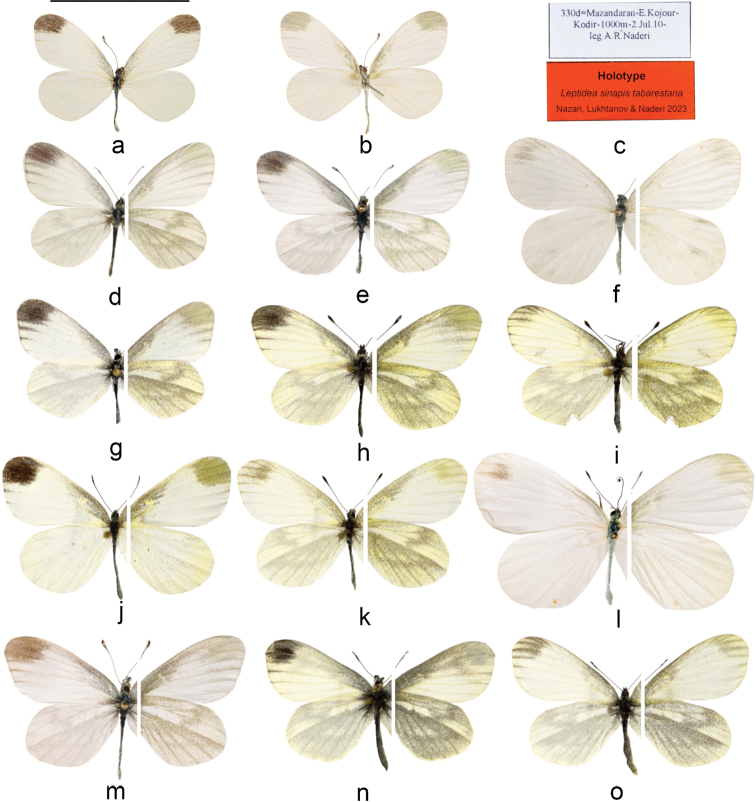
Adults **a–i**L.sinapisssp.tabarestana**j–o**L.sinapisssp.sinapis**a–c** holotype Mazandaran: Kojur (♂ ARPI-330d-001) **d** Golestan: Golestan forest (♂ ARPI-112j-001) **e** Gilan: Damash (♂ ARPI-524b-001) **f** Tehran: Laloon (♀ ARPI-408-001) **g** Mazandaran: Javaherdeh (♂ MR ZF 449) **h, i** Ardabil/Gilan: Talesh (♂♀ DNAwthLeptidea001–2) **j, k** Iran: E. Azerbaijan prov.: Kaleybar (♂ DNAwthLeptidea006, ♀-004) **l, m** Iran: E. Azerbaijan prov.: Arasbaran (♀ ARPI-479a [not barcoded], ♂ ARPI-456d-001) **n, o** Russia: Daghestan Republic (♂♀ DNAwthLeptidea010-11). Scale bar: 20 mm.

**Male** (Fig. 4a, b, d, e, g, h). Length of forewing 16–21 mm; ground colour pure white. ***First generation*** forewing upperside with a rectangular grey-black apical patch, veins v3 and v4 under this patch often covered with dark scales near the outer margin; forewing discal cell covered in grey scales that extend faintly along the costa towards the apex; a small dark patch near the base at the Inner margin. Hindwing upperside veins near the base of the wing covered with dark scales, otherwise without any other markings; the dark scales of the underneath show through. Forewing underside ground colour white with light yellowish-greenish tint at the apex, along the costa and at the discal cell except for a yellowish discoidal spot not covered in grey scales; all veins except v2 covered with dark scales at the outer half of the wing. Hindwing underside ground colour greenish-yellow covered in sparse grey scales; discal cell and space s5 lighter and covered in fewer dark scales; a faint postdiscal band broken into two sections: a costal S- shaped part and a lower postdiscal section in the form of a slightly curved streak. ***Second generation*** similar but grey scales on the underside highly reduced, sometimes completely absent.

**Female** (Fig. 4f, i). Length of forewing 19–23 mm; similar to male but bigger, forewing apex more rounded; apical dark patch highly reduced, sometimes absent.

***Male genitalia*** (Fig. 3 inset). Based on the eight dissections examined, the male genitalia appear similar to that of the nominotypical *sinapis*. The three elements of the male genitalia (phallus length, saccus length and vinculum width) measured for *L.s.tabarestana* ssp. nov. (PL: 1.47±0.07, SL: 0.60±0.06, VW: 0.71±0.04, n=8) were comparable to those of the nominotypical *L.sinapis* (PL: 1.60±0.08, SL: 0.63±0.04, VW: 0.79±0.05, n=48) (Suppl. material 2).

#### Diagnosis.

Morphologically inseparable from the nominotypical *L.sinapis*, however the new taxon is distinguishable from it only by COI barcodes. Unlike ssp. sinapis, which in Iran (East Azerbaijan province) is strictly limited to humid and damp forests or clearings, the new subspecies is found primarily in semi-humid or even semi-dry mountainous habitats.

#### Etymology.

The subspecies name is a reference to “Tabarestan”, the medieval name for the mountainous regions south of the Caspian coast in northern Iran and roughly corresponding to the modern-day province of Mazandaran, the type locality of *L.s.tabarestana* ssp. nov.

#### DNA barcode analysis.

The COI barcodes of *L.s.tabarestana* ssp. nov. fall within the Barcode Identification Number (BIN) of *L.sinapis* (BOLD:AAA6298), however they form a unique and distinct cluster that is on average 0.74% (range: 0.42%–1.76%) distant from all other *L.sinapis* (Fig. 1). Uncorrected *p*-distances are smaller than those between *L.sinapis* and *L.reali* (0.92%) or between *L.sinapis* and *L.juvernica* (1.97%) (Table 1). Since the topology of ML and Bayesian trees were similar, only the Bayesian tree is shown with ML bootstrap values plotted on the supported nodes. In both trees, the *L.s.tabarestana* ssp. nov. clade appeared as sister to all other *L.sinapis* samples with strong support (Fig. 1).

#### Karyotype.

**Figure 5.**
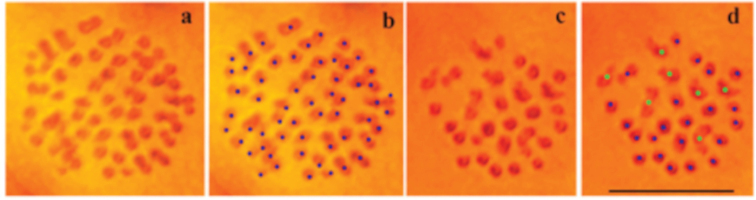
Karyotype of *Leptideasinapistabarestana* ssp. nov. **a, b** mitotic cell demonstrating ca 58 chromosomes (sample VLU396) **c, d**MII plate demonstrating 29 entities, 22 entities were interpreted as bivalents (shown by blue dots on Fig. 5d) and 7 entities were interpreted as trivalents (shown by green dots on Fig. 4d). Scale bar: 10 μm.

Of the 10 specimens studied, only two samples demonstrated metaphase plates suitable for counting the number of chromosomes. Such a low proportion of adult males with dividing cells is a common phenomenon in the genus *Leptidea* and has been noted previously (Lukhtanov et al. 2011). In the sample VLU396, in mitotic cells, the diploid number of chromosomes was determined to be approximately 2n=ca 58. An exact diploid number could not be determined due to numerous overlaps or contacts of chromosomes (Fig. 5).

The MI metaphase cells were not found in the studied individuals; however, MII metaphase plates were found in the sample VLU405. The MII plates demonstrated clear traces of the phenomenon for which we previously used the term *inverted meiosis* (Lukhtanov et al. 2018; 2020a, b). In this type of meiosis, heterozygosity for chromosomal fusions/fissions leads to the very specific chromosomal structures at the MII stage, when heterozygotes retain a configuration similar to that of trivalents. Such trivalent-like structures were observed at the MII stage in the sample U405 (shown in green in Fig. 5d). The number of such trivalent-like structures reached 7, while the total number of chromosome entities was n = 29. If these elements are interpreted as trivalents, then the diploid number can be estimated as 2n = 65. If these elements are bivalents, then the diploid chromosome number is 2n = 58. Thus, the preliminary haploid number of chromosomes can be estimated as n = 29–33.

Previously, a chromosome cline was found in *L.sinapis*, within which the diploid chromosome number gradually decreases from 2n = 106 in Spain to 2n = 56 in Sweden and in eastern Kazakhstan (Lukhtanov et al. 2011, 2018). Thus, the studied population from Mazandaran, Iran has an oriental variant of karyotype, that is, with a relatively low number of chromosomes. We were not able to study the karyotype from the Iranian Talesh; however, the karyotype of the population from Yardimli in Republic of Azerbaijan’s Talysh region was studied previously (Lukhtanov 1992). The latter population (Azerbaijani Talysh) demonstrated variation in the haploid chromosome number from n = 28 to n = 34 (Lukhtanov 1992), thus, similar to the Mazandaran population.

#### Distribution and ecology.

**Figure 6.**
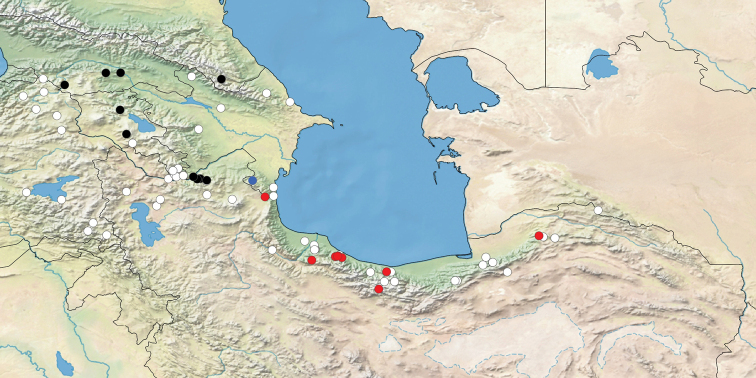
Distribution of *Leptideasinapis* in E Turkey, S Caucasus and N Iran. Black dots: barcoded *L.s.sinapis*; red dots: barcoded *L.s.tabarestana* ssp. nov.; blue dot: karyotyped sample from Yardamli in Republic of Azerbaijan’s Talysh region (most likely *L.s.tabarestana* ssp. nov.); white dots: non-barcoded material, data obtained from literature or personal collections.

So far, the presence of *L.s.tabarestana* ssp. nov. has been confirmed by DNA evidence only in northern Iran, in provinces of Ardabil, Gilan, Mazandaran, Tehran and Golestan (Fig. 6). Specimens from the Talysh mountains in Republic of Azerbaijan, across the border from Iranian Talesh region, show the same karyotype and possibly belong to ssp. tabarestana, however this remains to be further confirmed by DNA sequencing. In Turkmenistan, even though reports of *L.sinapis* from the Kopet Dagh mountains are as of yet unconfirmed (Tshikolovets 1998), these also likely belong to ssp. tabarestana.

**Figure 7.**
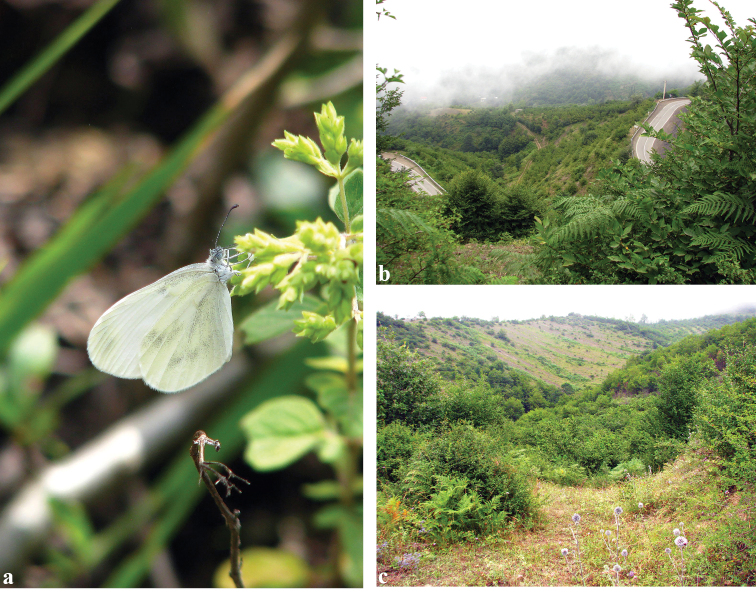
*Leptideasinapistabarestana* ssp. nov. **a** adult **b, c** habitat in Iran, Mazandaran Prov., Javaherdeh (Jirkooh), 24.VII.2011. Photos: V. Lukhtanov.

In the Iranian Talesh mountains, *L.s.tabarestana* ssp. nov. occurs approximately 100 km from the closest population of the nominotypical *L.sinapis* in Arasbaran region. The habitat of ssp. tabarestana is in the Alborz forest belt, in humid meadows, forest river banks, forest clearings, and sometimes gardens at mountain steppes from 1000 to 2000 m above sea level. Adults fly mostly in undisturbed or lightly-grazed habitats with lush of green vegetation (Fig. 7). The accompanying species include *Ochlodeshyrcana* (Christoph, 1893), *Pierisnapimazandarana* Eitschberger, 1987, *Lasiommataadrastiodes* (Bienert, [1870]), and *Maniolajurtina* (Linnaeus, 1758). It is normally found in two (or maybe three) generations, from April at lower altitudes to the end of September at higher altitudes. The early stages of *L.s.tabarestana* ssp. nov. are unknown, however adults are often seen near *Lathyrus* plants (AN, personal observation). Even though the larval host plant is likely among the herbaceous Fabaceae of the genera *Lathyrus*, *Vicia*, *Lotus* etc., it is as of yet unrecorded and thus it is unclear if ssp. tabarestana displays any host plant preferences different from the rest of populations of ssp. sinapis.

## ﻿Discussion

Reissinger (1989) recognized twelve subspecies of *L.sinapis* across its range, including *reali* and *juvernica*, both of which were later confirmed as separate species (Dincă et al. 2011, 2013). Since then, this complex has taken a central stage in efforts to understand the mechanisms of cryptic speciation in butterflies, and thus the idea of the existence of subspecies within *L.sinapis* seems to have slowly faded away. Modern taxonomic treatments of the group (e.g., Bozano et al. 2016) regard all populations of *L.sinapis* from Europe to NW Mongolia as a single entity, corresponding to the nominotypical subspecies. It occasionally flies in sympatry with closely related and extremely similar species of *Leptidea* across its range and can be separated from them only by DNA sequencing and analysis of karyotype or genitalia.

In a similar vein, in this study we found no single external morphological character or combination of characters that could reliably separate *L.s.tabarestana* ssp. nov. from the nominotypical *L.sinapis*. Individual variation in morphology observed within *L.s.tabarestana* ssp. nov. is not unexpected, as similar variation can also be seen in *L.s.sinapis*, as well as other species within the genus. In the Arasbaran mountains in NW Iran, where the nominotypical *L.sinapis* is found, individuals flying in colder slopes at high altitudes (1700–2000 m) tend to be smaller and darker, while those found in warmer forests at lower altitudes (1200–1400 m) are usually larger in size and have a lighter complexion.

Recent studies have estimated the age of the most recent common ancestor (MRCA) of *L.sinapis* at 1.5 mya, and for MRCA of *sinapis*+*juvernica* at 3 mya (Talla et al. 2017). The subsequent dispersal of *L.sinapis* eastward however appears to have occurred much later, either before or after the Last Glacial Maximum (LGM) (24,000 to 17,000 years ago) (Lukhtanov et al. 2011). During the Pleistocene, dense forests covered the entire northern Iran, from the northwest (Azerbaijan province) across the Alborz mountains and extending further into the northeast (Kopet Dagh); However, since the Last Glacial Maximum (LGM; 21 kya), the Alborz mountain range has been nearly entirely isolated from all other regions surrounding it. Subsequent decline in forest cover resulted in isolated refugia in parts of southern Caucasus as well as in northern Iran (Yousefi et al. 2015; Asadi et al. 2018; Parvizi et al. 2018; Liu et al. 2019; Saberi-Pirooz et al. 2020). With the likely extinction of intervening populations, the range of many butterflies adapted to this habitat – including the ancestral *L.sinapis* – became fragmented, resulting in the geographic and genetic isolation of *L.s.tabarestana* ssp. nov.

Presence of *Wolbachia* endosymbionts affecting mtDNA in *Leptidea* has been noted previously (e.g. Solovyev et al. 2015) and we cannot rule out that this may have had an effect on our results. Further studies are needed to confirm the presence of *L.s.tabarestana* ssp. nov. in the Republic of Azerbaijan and in Turkmenistan. Potential sympatric occurrence of the two entities in the intervening areas in NW Iran needs to be investigated. If the two are found to co-occur sympatrically and synchronically without geneflow, or other new information (e.g., karyotype, nuDNA, morphology etc.) comes to light that clearly signals the two taxa to be distinct at species level, the taxon *tabarestana* may be raised as bona species.

## Supplementary Material

XML Treatment for Leptidea sinapis ssp. tabarestana

## Supplementary materials

Supplementary material 1 Material examined and GenBank accessions Data type excel file https://binary.pensoft.net/file/846056This dataset is made available under the Open Database License (http://opendatacommons.org/licenses/odbl/1.0/). The Open Database License (ODbL) is a license agreement intended to allow users to freely share, modify, and use this Dataset while maintaining this same freedom for others, provided that the original source and author(s) are credited.Vazrick Nazari, Vladimir A. Lukhtanov, Alireza Naderi, Zdenek Faltýnek Fric, Vlad Dincă, Roger Vila

Supplementary material 2 Length of male genitalia components (phallus, saccus, vinculum) and ratios (PL/VW, SL/VW) among *Leptidea* specimens measured in this study Data type excel file https://binary.pensoft.net/file/846057This dataset is made available under the Open Database License (http://opendatacommons.org/licenses/odbl/1.0/). The Open Database License (ODbL) is a license agreement intended to allow users to freely share, modify, and use this Dataset while maintaining this same freedom for others, provided that the original source and author(s) are credited.Vazrick Nazari, Vladimir A. Lukhtanov, Alireza Naderi, Zdenek Faltýnek Fric, Vlad Dincă, Roger Vila
